# Genome-wide association study of stimulant dependence

**DOI:** 10.1038/s41398-021-01440-5

**Published:** 2021-06-29

**Authors:** Jiayi Cox, Richard Sherva, Leah Wetherill, Tatiana Foroud, Howard J. Edenberg, Henry R. Kranzler, Joel Gelernter, Lindsay A. Farrer

**Affiliations:** 1 Department of Medicine (Biomedical Genetics), Boston University School of Medicine, Boston, MA 02118 USA; 2 Departments of Medical and Molecular Genetics, Indiana University School of Medicine, Indianapolis, IN 46202 USA; 3 Biochemistry and Molecular Biology, Indiana University School of Medicine, Indianapolis, IN 46202 USA; 4 Perelman School of Medicine, University of Pennsylvania and VISN 4 MIRECC, Crescenz VAMC, Philadelphia, PA 19104 USA; 5 Departments of Psychiatry, Genetics and Neuroscience, Yale School of Medicine, New Haven, CT 06511 USA; 6 Department of Psychiatry, VA CT Healthcare Center, West Haven, CT 06516 USA; 7 Department of Neurology, Boston University School of Medicine, Boston, MA 02118 USA; 8 Department of Ophthalmology, Boston University School of Medicine, Boston, MA 02118 USA; 9 Department of Epidemiology, Boston University School of Public Health, Boston, MA 02118 USA; 10 Department of Biostatistics, Boston University School of Public Health, Boston, MA 02118 USA

**Keywords:** Clinical genetics, Addiction

## Abstract

Stimulant dependence is heritable, but specific genetic factors underlying the trait have not been identified. A genome-wide association study for stimulant dependence was performed in a discovery cohort of African- (AA) and European-ancestry (EA) subjects ascertained for genetic studies of alcohol, opioid, and cocaine use disorders. The sample comprised individuals with DSM-IV stimulant dependence (393 EA cases, 5288 EA controls; 155 AA cases, 5603 AA controls). An independent cohort from the family-based Collaborative Study on the Genetics of Alcoholism (532 EA cases, 7635 EA controls; 53 AA cases, AA 3352 controls) was used for replication. One variant in *SLC25A16* (rs2394476, *p* = 3.42 × 10^−10^, odds ratio [OR] = 3.70) was GWS in AAs. Four other loci showed suggestive evidence, including *KCNA4* in AAs (rs11500237, *p* = 2.99 × 10^−7^, OR = 2.31) which encodes one of the potassium voltage-gated channel protein that has been linked to several other substance use disorders, and *CPVL* in the combined population groups (rs1176440, *p* = 3.05 × 10^−7^, OR = 1.35), whose expression was previously shown to be upregulated in the prefrontal cortex from users of cocaine, cannabis, and phencyclidine. Analysis of the top GWAS signals revealed a significant enrichment with nicotinic acetylcholine receptor genes (adjusted *p* = 0.04) and significant pleiotropy between stimulant dependence and alcohol dependence in EAs (*p*_*adj*_ = 3.6 × 10^−3^), an anxiety disorder in EAs (*p*_*adj*_ = 2.1 × 10^−4^), and ADHD in both AAs (*p*_*adj*_ = 3.0 × 10^−33^) and EAs (*p*_*adj*_ = 6.7 × 10^−35^). Our results implicate novel genes and pathways as having roles in the etiology of stimulant dependence.

## Introduction

Amphetamines have been used to treat a variety of conditions including asthma, obesity, and attention-deficit/hyperactivity disorder (ADHD)^1^. Amphetamines and other stimulants increase alertness and physical and mental performance and reduce drowsiness. The mechanism by which stimulants exert these effects appears to involve the increase in the level of dopamine (DA) in the striatum that results from their competitive inhibition of DA uptake, which facilitates DA release from synaptic vesicles, and their promotion of reverse transport of DA into the synaptic cleft^2,3^. In some individuals, amphetamines induce pleasurable effects. However, misuse of stimulants saturates DA receptors, disrupts the normal production of DA, and may lead to severe pathophysiological effects, including tachycardia and myocardial infarction, withdrawal-related outcomes such as anxiety, depression, and psychosis^3^.

The misuse of amphetamines is a public health problem. Emergency room visits related to stimulant abuse increased from 2303 in 2004 to 17,272 in 2011^4^. In 2015, there were ∼5.3 million non-medical users of prescription stimulants among individuals age 12 and older in the United States^5^. A meta-analysis of published neuroimaging data in individuals meeting DSM-IV criteria for stimulant dependence showed reduced gray matter in prefrontal cortical regions that are associated with self-regulation and self-awareness^6^.

Family and twin studies have shown that the risk of stimulant use disorder is proportional to the degree of relatedness to an affected relative^1,7^. The heritability of stimulant use disorder (excluding cocaine) has been estimated to be 0.40–0.42^8,9^. Although a genome-wide association study (GWAS) of methamphetamine dependence yielded no significant findings, the sample of 580 individuals was likely insufficient to detect associations with variants of modest effect^10^. We performed a GWAS for stimulant dependence in a discovery sample of 5681 individuals of European ancestry (EA) and 5758 of African ancestry (AA) and, after testing the top-ranked findings in an independent dataset with 3405 AA and 8185 EA individuals, identified two genome-wide significant (GWS) associations. These results provide insight into the biological basis of stimulant dependence.

## Subjects and methods

### Participants and diagnostic procedures

The discovery sample was derived from the Yale-Penn sample, a cohort of 11,439 participants (5758 AAs and 5681 EAs) recruited through treatment centers and advertisements for genetic studies of cocaine, opioid or alcohol dependence^11^. All participants were interviewed using the Semi-Structured Assessment for Drug Dependence and Alcoholism (SSADDA)^12^, which we have previously shown to be reliable with respect to both diagnoses and diagnostic criteria^13,14^, to derive lifetime diagnoses for dependence on these and other substances and other major psychiatric disorders. DSM-IV dependence on stimulants (including amphetamine-related substances) was assessed using information from the SSADDA. Individuals who had a dependence on other stimulants (including cocaine and caffeine) were not considered as stimulant dependent in order to minimize genetic heterogeneity in the outcome and detect variants specifically relevant to dependence on amphetamines and closely related stimulants. Additional details of participant recruitment and assessment are reported elsewhere^11,15^. After excluding participants with missing stimulant use or basic demographic information, the remaining sample consisted of 614 small nuclear families (1355 total participants) and 10,084 unrelated individuals. An independent sample consisting of 532 EA cases, 7635 EA controls, 53 AA cases, and AA 3352 controls was selected from the Collaborative Study on the Genetics of Alcoholism (COGA)^16^ for replication. Diagnoses in the COGA sample were made using the SSAGA, a semi-structured interview from which the SSADDA was derived^17^. Characteristics of stimulant-dependent cases and controls in the discovery and replication datasets are shown in Table 1. This study was approved by the Institutional Review Boards at all participating sites. Data were analyzed between September 2017 and October 2019.

**Table 1:** Sample characteristics.

Stage	Dataset	Group	African Americans		European Ancestry	
			Female/total	Age µ (SD)	Female/total	Age µ (SD)
Discovery	**Yale-Penn1**	Case	32/101	47.0 (7.8)	73/169	40.5 (10.0)
		Control	1427/2986	40.8 (9.0)	603/1394	37.7 (11.0)
	**Yale-Penn2**	Case	10/38	48.1 (10.9)	48/136	42.0 (13.2)
		Control	684/1617	40.6 (11.0)	615/1461	39.1 (13.0)
	**Yale-Penn3**	Case	6/16	49.8 (11.0)	20/88	41.4 (11.4)
		Control	486/1000	40.5 (11.4)	1,219/2423	40.5 (14.6)
Replication	**COGA**	Case	21/53	40.6 (8.5)	222/532	36.8 (9.3)
		Control	1783/3299	32.7 (12.2)	3798/7103	36.8 (15.2)

### Genotyping, imputation, quality control, and population substructure analysis

As described previously^11^, specimens from participants in the discovery sample were genotyped using one of three genome-wide SNP arrays: the Illumina HumanOmni1-Quad v1.0 microarray containing 988,306 autosomal SNPs (Yale-Penn 1), the Illumina Infinium Human Core Exome microarray containing 265,919 exonic SNPs and approximately 240,000 tagging SNPs (Yale-Penn 2), and the Illumina Multi-ethnic Global Array containing 1,779,819 markers representing five major populations (Yale-Penn 3). Genotyping was performed at the Yale Center for Genome Analysis, except for a group of 2538 samples (1784 AAs and 754 EAs) that were genotyped at the Center for Inherited Disease Research. Quality control of genotype data was performed as previously described^18^. Briefly, individuals with a call rate < 98% and variants with minor allele frequency (MAF) < 1% were excluded. Pairwise identity-by-decent (IBD) was calculated with PLINK^19^ to determine genetic relatedness among individuals in the sample and individuals with a pairwise IBD estimate > 25% were assigned to the same family. Self-reported males with X chromosome heterozygosity > 20% and self-reported females with X chromosome heterozygosity < 20% were excluded. Population substructure in the entire sample was evaluated by analysis of the principal components (PCs) of ancestry using Eigensoft^20^ and the multi-ethnic 1000 Genome reference panel for comparison. Individuals were classified as AA or EA according to the reference panel population to which they were more closely matched. SNP genotype imputation was performed separately in AAs and EAs using the March 2012 1000 Genomes reference panel (1000 Genomes Project, 2012; http://www.1000genomes.org/) and Minimac3^21^ implemented on the Michigan imputation server (https://imputationserver.sph.umich.edu). Genotyping, QC, and imputation procedures for the COGA dataset are described elsewhere^22^. Analysis was limited to SNPs with an imputation quality score > 0.8 and MAF > 0.03.

### Genome-wide association analyses

Association of the DSM-IV diagnosis of stimulant dependence was evaluated using logistic regression models that were solved with generalized estimating equations to correct for correlations among related individuals. Models included covariates for age, sex, and the first five PCs. Association tests were performed separately within each population group and within each genotyping platform to account for batch effects. The association test results were corrected for genomic inflation (λ) and combined across population and batch groups via inverse variance meta-analysis implemented in the program METAL^23^. We ignored results for variants whose heterogeneity *p*-values from the meta-analysis were less than 1.4 × 10^−6^ in AAs or 3.3 × 10^−9^ in EAs (different thresholds were used given the sample size difference across populations) implying inconsistency across datasets. The *p* -value threshold was set at 5.0 × 10^−8^ for GWS. A suggestive significance level was set at 5.0 × 10^−6^, and replication was sought for variants that passed this threshold. Association testing in the replication dataset was performed using the same covariates as in the discovery sample in regression models implemented in geepack (https://cran.r-project.org/web/). Results for the discovery and replication datasets were combined using the inverse variance meta-analysis as described above.

### Assessment of SNP effects on gene expression

SNPs that surpassed the significance threshold of *p* = 1.0 × 10^−6^ in the GWAS discovery dataset were assessed for their potential to affect gene expression using the information in the Genotype-Tissue Expression Portal (GTEx)^24^ (http://www.gtexportal.org) and Braineac^25^ (http://www.braineac.org/). GTEx links SNP genotype to gene expression in multiple human tissues, whereas Braineac incorporates expression data for multiple brain regions derived from 130 individuals from the UK Brain Expression Consortium (UKBEC) and contains expression-altering SNP information for each brain region.

### Pleiotropy analyses

Because > 70% of persons with stimulant use disorder have comorbid alcohol or cannabis use disorders and more than one-third have anxiety disorder^26^, and amphetamine-related medications are used to treat attention deficit hyperactivity disorder (ADHD)^27^, we investigated the possibility of pleiotropy using GWAS summary statistics that were available in 2017 from the Psychiatric Genetics Consortium on LD Hub^28^ for ADHD (in a predominantly EA sample)^29^, alcohol dependence (in a trans-ancestral sample)^30^, and anxiety disorder (in an EA sample)^31^. Pleiotropy analyses were performed using a mixture model implemented in the Genetic Analysis Incorporating Pleiotropy and Annotation (GPA) software^32^. Parameters were estimated using GPA’s efficient expectation-maximization algorithm, wherein associated SNPs were modeled with a β [α, 1] distribution and unassociated SNPs with a uniform [0, 1] distribution. A likelihood ratio test was applied to determine the significance of pleiotropy between disorders based on an evaluation of the entire genome as well as individual SNPs.

### Pathway analysis

Biological pathways were evaluated using the Enrichr software^33^ (http://amp.pharm.mssm.edu/Enrichr/), which considers gene sets derived from population-specific GWAS results and canonical pathways culled from multiple sources (e.g., membership of genes in pathway databases^34^, protein-protein interaction network data extracted from literature, disease databases^35,36^, gene expression profiling^24,37^. Variants were mapped to genes using SNPEff^38^ and the smallest *p* -value within each gene was corrected by the effective number of SNPs tested in that gene according to the Li and Ji method^39^. We set the corrected significance threshold at *p* < 0.001 in order to obtain 200–300 genes for subsequent pathway analyses. This yielded a list of 235 genes from AAs and EAs. Enrichr uses Fisher’s exact test to calculate an enrichment score. The test for each pathway was computed by comparing its observed rank with the expected rank using multiple random input gene lists.

## Results

### GWAS findings

**Fig. 1:**
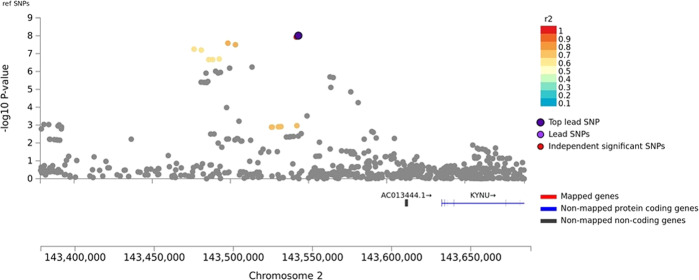
Association of stimulant dependence with SNPs located between *LRP1B* and *KYNU* in the African American discovery sample. SNPs are color-coded according to the correlation coefficient (*r*^*2*^) in the 1000 Genomes African reference panel with the top-ranked SNP, rs6721393. Rs6721393 was nearly genome-wide significant (*P* = 3.13 × 10^−7^) after meta-analysis with the replication sample.

**Fig. 2:**
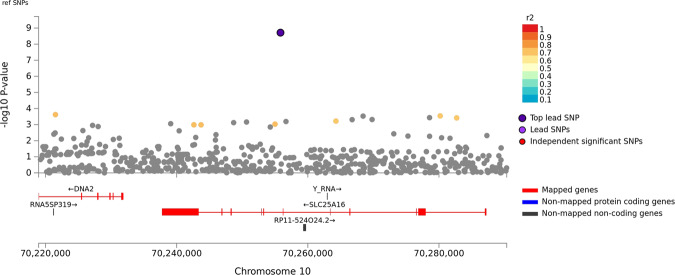
Association of stimulant dependence with SNPs located in the *SLC25A16* region in the African American discovery sample. SNPs are color-coded according to the correlation coefficient (*r*^*2*^) in the 1000 Genomes African reference panel with the top-ranked SNP, rs2394476. Rs2394476 was genome-wide significant (*P* = 1.22 × 10^−9^) after meta-analysis with the replication sample.

**Fig. 3:**
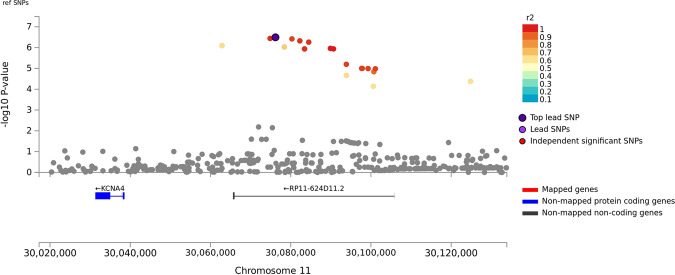
Association of stimulant dependence with SNPs located in the *KCNA4* region in the African American discovery sample. SNPs are color-coded according to the correlation coefficient (*r*^*2*^) in the 1000 Genomes African reference panel with the top-ranked SNP, rs11500237 located 93 kb upstream of *KCNA4*. Rs11500237 was nearly genome-wide significant (*P* = 2.99 × 10^−7^) after meta-analysis with the replication sample.

**Fig. 4:**
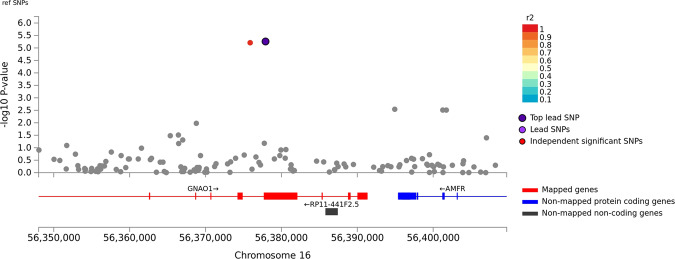
Association of stimulant dependence with SNPs located in the *GNAO1* region in the African American discovery sample. SNPs are color-coded according to the correlation coefficient (*r*^*2*^) in the 1000 Genomes African reference panel with the top-ranked SNP, rs116441240. Rs116441240 was nearly genome-wide significant (*P* = 1.09 × 10^−7^) after meta-analysis with the replication sample.

**Fig. 5:**
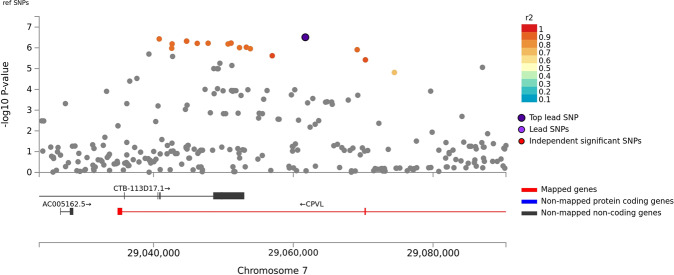
Association of stimulant dependence with SNPs located in the *CPVL* region in the combined African American and European ancestry discovery sample. SNPs are color-coded according to the correlation coefficient (*r*^*2*^) in the 1000 Genomes combined European and African reference panels with the top-ranked SNP, rs116441240. Rs116441240 was nearly genome-wide significant (*P* = 3.05 × 10^−7^) after meta-analysis with the replication sample.

There was minor *p*-value inflation in the AA (λ = 1.02), EA (λ = 1.038), and combined (λ = 1.041) discovery samples (Fig. S1). Several variants showed evidence for association at the genome-wide or suggestive significance level (Table 2, Fig. S2). In AAs, the association with SNP rs2394476 located between *LRP1B* (443 kb upstream) and *KYNU* (93 kb upstream) surpassed the genome-wide cutoff (*p* = 1.19 × 10^−8^, OR = 2.12) and had the same effect direction in all subsets of the data (Fig. 1). GWS evidence was also obtained with *SLC25A16* SNP rs2394476 (*p* = 3.42 × 10^−10^, OR = 3.7, Fig. 2). Variants in two other regions were highly significant (*p* < 1.0 × 10^−5^) in AAs: rs11500237 located 37 kb from *KCNA4* (*p* = 3.21 × 10^−7^, OR = 2.56, Fig. 3) and rs116441240 located in *GNAO1* (*p* = 5.51 × 10^−6^, OR = 2.73, Fig. 4). No SNPs approached the GWS level in EAs. There was strong evidence for association with intronic *CPVL* variant rs11764430 in both AAs (*p* = 1.38 × 10^−4^) and EAs (*p* = 4.64 × 10^−4^), and this SNP was nearly GWS in the combined AA and EA discovery datasets (*p* = 3.10 × 10^−7^, OR = 1.60, Fig. 5).

**Table 2:** Results (*p* < 5.0 × 10^−7^) for GWAS of stimulant dependence in the discovery dataset or combined discovery and replication datasets according to population ancestry. Only the most significant SNP at each locus is shown.

A. *African Americans*																
Chr: position	SNP ID	Locus	MA	Discovery				Replication				Total				
				MAF case	MAF control	OR	*P*-value	MAF case	MAF control	OR	*P*-value	OR [95% C.I.]	*P*-value	Dir.^a^		
2:143542131	rs6721393	*LRP1B-KYNU*	C	0.19	0.16	2.12	1.19 × 10^−8^	0.16	0.86	0.15	0.61	1.86 [1.47–2.36]	3.13 × 10^−7^	+++-		
10:70255832	rs2394476	*SLC25A16*	G	0.1	0.03	3.70	3.42 × 10^−10^	NA	NA	NA	NA	3.70 [2.26–6.06]	3.42 × 10^−10^	+x+x		
11:30076200	rs11500237	*KCNA4*	C	0.12	0.06	2.56	3.21 × 10^−7^	0.08	0.05	1.54	0.24	2.31 [1.64–3.14]	2.99 × 10^−7^	++++		
16:56377908	rs116441240	*GNAO1*	T	0.11	0.04	2.73	5.51 × 10^−6^	0.10	0.04	2.53	0.0065	2.66 [1.64–3.91]	1.09 × 10^−7^	++++		

B. *Combined populations*																
Chr: position	SNP ID	Locus	MA	Discovery		AA replication				EA replication				Total		
				OR	*P*-value	MAF case	MAF control	OR	*P*-value	MAF case	MAF control	OR	*P*-value	OR	*P*-value	Dir.^a^
7:29061724	rs11764430	*CPVL*	T	1.49	3.10 × 10^−7^	0.26	0.21	1.60	0.0024	0.14	0.13	1.10	0.34	1.35 [1.22–1.54]	3.05 × 10^−7^	++++++++

In (A), the first three symbols are for batches of the discovery dataset analyzed separately, and the fourth symbol is for the replication dataset. In (B), the first three symbols are for batches of the AA discovery dataset analyzed separately, the next three symbols are for batches of the EA discovery dataset analyzed separately, and the last two symbols are for the replication AA and EA datasets, respectively.

*MA* minor allele, *MAF* minor allele frequency, *OR* odds ratio, *NA* result not available.

^a^ Effect direction: + = OR > 1, - = OR < 1, x = no result available.

In the discovery GWAS, 59 SNPs (41 in AAs, 16 in EAs, and 2 in the meta-analysis) surpassed the suggestive threshold (*p* < 5.0 × 10^−6^) and were tested in the replication phase (Table S1). Results for the GWS *SLC25A16* SNP in the replication sample were unavailable due to a very small minor allele count. The finding with the *GNAO1* SNP that was nearly GWS in the discovery sample was replicated (*p* = 0.0065) and nearly GWS in the combined sample (*p* = 1.09 × 10^−7^, OR = 2.66, Table 2). In contrast, the association with the *LRP1B-KYNU* SNP that was GWS in the discovery sample was not confirmed in the replication sample but was still highly significant in the combined sample (*p* = 3.13 × 10^−7^). The associations with the *KCNA4* and *CPLV* SNPs were slightly more significant when combined with the replication datasets (*p* = 2.99 × 10^−7^ and *p* = 3.05 × 10^−7^, respectively), noting that *the CPVL* SNP was significant in the AA replication sample (*p* = 0.0024) and the effect direction was the same across all eight datasets. Two SNPs in Table 2 had significant eQTL effects in GTEx: rs11500237 on chromosome 11 near *KCNA4* is a significant eQTL for ADP ribosylation factor like GTPase 14 effector protein (*ARL14EP*) in prostate tissue (*p* = 2.3 × 10^−6^), and rs11764430 in CVPL significantly alters the expression of two uncharacterized transcripts (*p* = 8.3 × 10^−7^, *p* = 3.6 × 10^−6^).

In light of the potentially shared physiological pathways between nicotinic receptors and methamphetamine, we re-analyzed the discovery GWAS data including the Fagerstrom Test of Nicotine Dependence (FTND) score as a covariate in the regression model. No additional significant associations with stimulant dependence were identified, however, nor did the top findings change meaningfully.

### Genetic correlation of stimulant dependence with other psychiatric disorders

Table 3 shows that in AAs, stimulant dependence was significantly but modestly genetically correlated with alcohol dependence (r^2^ = 0.11, *p* = 8.0 × 10^−16^), ADHD (r^2^ = 0.05, *p* = 3.5 × 10^−5^), and anxiety disorder (r^2^ = 0.03, *p* = 9.2 × 10^−3^). In EAs, the correlation with both alcohol dependence and ADHD was nearly double the magnitude and substantially more significant (r^2^ = 0.20, *p* = 7.2 × 10^−55^ and r^2^ = 0.10, *p* = 1.5 × 10^−14^, respectively) than in AAs; these differences could have been due to the ancestry of the reference GWAS sample. The pleiotropy analysis showed that the variants associated with stimulant dependence also affected the risk of alcohol dependence (adjusted *p* = 3.6 × 10^−3^) and anxiety (adjusted *p* = 2.1 × 10^−4^) in EAs but not AAs. Although pleiotropy was observed for stimulant dependence and ADHD in both AAs (adjusted *p* = 3.0 × 10^−33^) and EAs (adjusted *p* = 6.7 × 10^−35^), no individual variants showed significant pleiotropic effects on stimulant dependence and any of the other disorders after multiple testing correction.

**Table 3:** Genetic correlation (*r*_*g*_) and pleiotropy between stimulant dependence and other psychiatric disorders by population group.

Trait	African American			European Ancestry		
	*r*_*g*_	Correlation *p*-value	Pleiotropy *p*_*adj*_	*r*_*g*_	Correlation *p*-value	Pleiotropy *p*_*adj*_
Alcohol dependence	0.11	8.0E−16	1.1E−01	0.20	7.2E−55	3.6E−03
ADHD	0.053	3.5E−05	3.0E−33	0.10	1.5E−14	6.7E−35
Anxiety	0.033	9.2E−03	8.9E−01	−0.002	8.8E−01	2.1E−04

### Pathway analyses

After correction for multiple testing, analyses that were seeded with the 235 top-ranked genes *(p* < 0.001) identified in the GWAS revealed nicotinic acetylcholine receptor activity (nAChR) as the only significant pathway (adjusted *p* = 3.6 × 10^−2^). Among the genes in this pathway, *CHRNA3, CHRNB4*, and *CHRNA5* contained SNPs that were associated with stimulant dependence in the combined population (Table 4).

**Table 4:** Top-ranked associations of stimulant dependence with individual variants in each of three adjacent nicotinic acetylcholine receptor genes observed in the discovery GWAS in African American and European ancestry groups.

Population	Gene	SNP ID	Chr: position	*p*-value
African American	*CHRNA3*	rs16969968	15:78882925	5.6E−04
	*CHRNA5*	rs2036527	15:78854706	6.6E−02
	*CHRNB4*	rs190245674	15:78913207	1.1E−02
European Ancestry	*CHRNA3*	rs16969968	15:78882925	2.9E−06
	*CHRNA5*	rs12900711	15:78853998	1.2E−05
	*CHRNB4*	rs12907519	15:78913353	3.8E−06

## Discussion

To our knowledge, this is the first study to report a GWS association for dependence on stimulants other than cocaine. We identified a SNP at *SLC25A16* that was significantly associated with the trait in AAs. Near-GWS associations were also identified in AAs with SNPs in *GNAO1*, between *LRP1B* and *KYNU*, and near *KCNA4*. A *CPVL* SNP was also nearly GWS with evidence in both AAs and EAs. We also identified significant enrichment among suggestively associated SNPs for genes in the nicotinic acetylcholine receptor activity pathway and a genetic underpinning for stimulant dependence shared with ADHD and alcohol dependence.

Several of the top-ranked variants are mapped to loci that were not previously implicated in substance use and other psychiatric disorders. *KCNA4* encodes a potassium voltage-gated channel protein. Potassium voltage-gated channels have been implicated in opioid dependence^18^, the long-acting narcotic analgesic narcotic l-alpha-acetylmethadol^40^, and alcohol-preferring rats treated with lamotrigine^41^. Mutations in *GNAO1*, which encodes the alpha subunit of the G-alpha heterotrimeric G-protein signal-transducing complex, cause early-onset epileptic encephalopathy and severe developmental delay^42–44^. *GNAO1* expression is upregulated in a mouse model of morphine dependence, and the knock-down of the gene in these animals led to reduced opioid withdrawal behaviors^45^. Although the exact function of the enzyme encoded by *CPVL* has not been confirmed, its expression is upregulated in the postmortem prefrontal cortex from users of cocaine, cannabis, and phencyclidine^46^. The *CPVL* variant associated with stimulant dependence, rs11764430, is an eQTL for *CHN2*, which regulates axonal pruning via the Rac-GTPase system^47^ and plays a pivotal role in axon guidance. A *CHN2* variant has been associated with smoking behavior^48^. Significant association of a quantitative serum measure of methylation of the *CHN2* promoter with methamphetamine dependence was observed in a Chinese sample^49^.

The role of *SLC25A16* in stimulant dependence is unclear. This gene encodes a transporter of dephospho-coenzyme A (CoA) across the inner mitochondrial membrane^50^. Interestingly, kynureninase, the enzyme encoded by one of the other top-ranked loci in this study (*KYNU*), catalyzes the cleavage of kynurenine. Kynurenines may play a role in schizophrenia^51^ and one of the kynurenine metabolites, pantethine, is a precursor in the formation of CoA^52^, Thus, our genetic findings suggest a potential involvement of CoA metabolism in stimulant dependence. This idea is supported by a metabolomics study pointing to an increased energy demand caused by amphetamine and a commensurate increase in the number of fatty acids^53^. Fatty acid catabolism produces energy (adenosine triphosphate, ATP) by mitochondrial beta-oxidation yielding acetyl-CoA.

The nAChR system is part of the brain reward circuitry that mediates the rewarding effect of amphetamine drugs by facilitating the release of dopamine^54,55^, and plays a key role in drug self-administration^56^. Repeated exposure to methamphetamine inhibited the corticostriatal release of dopamine similar to the classic nAChR agonist nicotine, an effect reversed by methamphetamine re-administration^57^. The *CHRNA3-CHRNA5-CHRNB4* gene cluster of nAChRs has been associated consistently with nicotine dependence^17^ and multiple smoking behaviors^58,59^.

Our pleiotropy analysis showed genetic overlap between stimulant dependence and alcohol use disorder, anxiety, and ADHD. Stimulants are widely used as a treatment for ADHD^18^, however there is disagreement about whether prescribing amphetamine for ADHD increases the risk of substance abuse in adulthood^60–62^. Studies of an ancestrally diverse set of cohorts (Thai, Malaysian, American, Chinese, and Australian)^25,63–67^ have demonstrated high comorbidity between psychiatric disorders including major depressive disorder^26,64^, anxiety disorder^26,65^ and alcohol use disorder^26,65^ in amphetamine-informative cohorts. It is not surprising that in our study individual variants associated with stimulant dependence also affected the risk of alcohol dependence and anxiety in EAs but not AAs because the GWAS summary data for these other disorders were derived primarily from EA cohorts.

Our study has several limitations. First, although all of the most significant variants are supported by surrounding SNPs, SNPs located at the association peak for several of the top loci are located in intergenic regions for which there is little evidence of a functional impact. Second, a high proportion of stimulant-dependent cases in the discovery and replication cohorts are dependent on other substances, so our results might not generalize to all individuals with amphetamine-related stimulant dependence. Third, the inclusion of both exposed and unexposed controls in this study may have reduced power due to misclassification; i.e. come controls might carry significant risk for stimulant dependence but were not exposed. Fourth, the number of stimulant-dependent cases is small for a GWAS and, not surprisingly, several associated variants have a large effect size. This is particularly true of the AA sample. Fifth, it is possible that some of our results were diluted because the interview instrument does not distinguish the use of methamphetamine from several other stimulant drugs. Finally, the significant enrichment for nicotinic acetylcholine receptor genes in the pathway analysis may be the result of either comorbidity and/or pleiotropy with nicotine dependence. To explore this possibility, we conducted a secondary association analysis for the top-ranked results using models that included a covariate for nicotinic dependence severity measured by the number of DSM-IV criteria endorsed. The results were not meaningfully different from those of the primary analyses.

We found an association of stimulant dependence with novel risk genes and genes that were previously identified as risk factors for other addiction traits. Post-GWAS eQTL and pathway analyses provide insight into the biological mechanisms that contribute to amphetamine dependence. In addition, our results suggest the presence of a shared genetic basis for stimulant dependence and other psychiatric traits.

## Supplementary information

Supplementary Figures

Supplementary Table 1

## References

[1] Hart AB, de Wit H, Palmer AA (2012). Genetic factors modulating the response to stimulant drugs in humans. Curr. Top. Behav. Neurosci..

[2] Seiden LS, Sabol KE, Ricaurte GA (1993). Amphetamine: effects on catecholamine systems and behavior. Annu. Rev. Pharmacol. Toxicol..

[3] Calipari ES, Ferris MJ (2013). Amphetamine mechanisms and actions at the dopamine terminal revisited. J. Neurosci..

[4] Substance Abuse and Mental Health Services Administration (SAMHSA), D.A.W.N., 2011: *National Estimates of Drug-Related Emergency Department Visits*. (Substance Abuse and Mental Health Services Administration, 2013).

[5] Hughes, A. et al. Prescription drug use and misuse in the United States: results from the 2015 national survey on drug use and health. *NSDUH Data Rev*. https://www.samhsa.gov/data/sites/default/files/NSDUH-FFR2-2015/NSDUH-FFR2-2015.htm (2016).

[6] Ersche KD, Williams GB, Robbins TW, Bullmore ET (2013). Meta-analysis of structural brain abnormalities associated with stimulant drug dependence and neuroimaging of addiction vulnerability and resilience. Curr. Opin. Neurobiol..

[7] Fergusson DM, Horwood LJ, Lynskey MT, Madden PA (2003). Early reactions to cannabis predict later dependence. Arch. Gen. Psychiatry.

[8] Goldman D, Oroszi G, Ducci F (2005). The genetics of addictions: uncovering the genes. Nat., Rev. Genet..

[9] Kendler KS, Gardner C, Jacobson KC, Neale MC, Prescott CA (2005). Genetic and environmental influences on illicit drug use and tobacco use across birth cohorts. Psychol. Med..

[10] Uhl GR (2008). Genome-wide association for methamphetamine dependence convergent results from 2 samples. Arch. Gen. Psychiatry.

[11] Sherva R (2016). Genome wide association study of cannabis dependence severity, novel risk variants, and shared genetic risks. JAMA Psychiatry.

[12] Pierucci-Lagha A (2005). Diagnostic reliability of the semi-structured assessment for drug dependence and alcoholism (SSADDA). Drug. Alcohol. Depend..

[13] Pierucci-Lagha A (2007). Reliability of DSM-IV diagnostic criteria using the semi-structured assessment for drug dependence and alcoholism (SSADDA). Drug. Alcohol. Depend..

[14] Malison RT (2011). Inter-rater reliability and concurrent validity of DSM-IV opioid dependence in a Hmong isolate using the Thai version of the semi-structured assessment for drug dependence and alcoholism (SSADDA). Addict. Behav..

[15] Zhou H (2017). Genetic risk variants associated with comorbid alcohol dependence and major depression. JAMA Psychiatry.

[16] Edenberg H (2005). Description of the data from the collaborative study on the genetics of alcoholism (COGA) and single-nucleotide polymorphism genotyping for genetic analysis workshop 14. BMC Genet..

[17] Bucholz KK (1994). A new, semi-structured psychiatric interview for use in genetic linkage studies: a report on the reliability of the SSAGA. J. Stud. Alcohol..

[18] Gelernter J (2014). Genomewide association study of opioid dependence and related traits: multiple associations mapped to calcium and potassium pathways. Biol. Psychiatry.

[19] Purcell S (2007). PLINK: a toolset for whole-genome association and population-based linkage analysis. Am. J. Hum. Genet..

[20] Price AL (2006). Principal components analysis corrects for stratification in genome-wide association studies. Nat. Genet..

[21] Howie B, Fuchsberger C, Stephens M, Marchini J, Abecasis GR (2012). Fast and accurate genotype imputation in genome-wide association studies through pre-phasing. Nat. Genet..

[22] Lai D (2019). Genome-wide association studies of alcohol dependence, DSM-IV criterion count and individual criteria. Genes. Brain. Behav..

[23] Willer CJ, Li Y, Abecasis GR (2010). METAL: fast and efficient meta-analysis of genomewide association scans. Bioinformatics.

[24] Carithers LJ (2015). A novel approach to high-quality postmortem tissue procurement: The GTEx Project. Biopreserv. Biobank..

[25] Ramasamy A (2014). Genetic variability in the regulation of gene expression in ten regions of the human brain. Nat. Neurosci..

[26] Sara G (2012). Stimulant use disorders: characteristics and comorbidity in an Australian population sample. Aust. N. Z. J. Psychiatry.

[27] Cortese S (2018). Comparative efficacy and tolerability of medications for attention-deficit hyperactivity disorder in children, adolescents, and adults: a systematic review and network meta-analysis. Lancet Psychiatry.

[28] Zheng J (2017). LD Hub: a centralized database and web interface to perform LD score regression that maximizes the potential of summary level GWAS data for SNP heritability and genetic correlation analysis. Bioinformatics.

[29] Martin J (2018). A genetic investigation of sex bias in the prevalence of attention-deficit/hyperactivity disorder. Biol. Psychiatry.

[30] Walters RK (2018). Trans-ancestral GWAS of alcohol dependence reveals common genetic underpinnings with psychiatric disorders. Nat. Neurosci..

[31] Otowa T (2016). Meta-analysis of genome-wide association studies of anxiety disorders. Mol. Psychiatry.

[32] Chung D, Yang C, Li C, Gelernter J, Zhao H (2014). GPA: a statistical approach to prioritizing GWAS results by integrating pleiotropy and annotation. PLoS. Genet..

[33] Kuleshov MV (2016). Enrichr: a comprehensive gene set enrichment analysis web server 2016 update. Nucleic Acids Res..

[34] Kanehisa M, Goto S (2000). KEGG: kyoto encyclopedia of genes and genomes. Nucleic Acids Res..

[35] Pletscher-Frankild S, Palleja A, Tsafou K, Binder JX, Jensen LJ (2015). DISEASES: text mining and data integration of disease-gene associations. Methods.

[36] Amberger JS, Bocchini CA, Scott AF, Hamosh A (2019). OMIM.org: leveraging knowledge across phenotype-gene relationships. Nucleic Acids Res..

[37] Uhlen M (2017). A pathology atlas of the human cancer transcriptome. Science.

[38] Cingolani P (2012). A program for annotating and predicting the effects of single nucleotide polymorphisms, SnpEff: SNPs in the genome of Drosophila melanogaster strain w1118; iso-2; iso-3. Fly.

[39] Li J, Ji L (2005). Adjusting multiple testing in multilocus analyses using the eigenvalues of a correlation matrix. Heredity.

[40] Kang J, Chen XL, Wang H, Rampe D (2003). Interactions of the narcotic l-alpha-acetylmethadol with human cardiac K+ channels. Eur. J. Pharm..

[41] Zalewska-Kaszubska, J. et al. Voluntary alcohol consumption and plasma beta-endorphin levels in alcohol preferring rats chronically treated with lamotrigine. *Physiol. Behav.* **139**, 7–12 (2015). 10.1016/j.physbeh.2014.11.026 25449391

[42] Danti FR (2017). GNAO1 encephalopathy: broadening the phenotype and evaluating treatment and outcome. Neurol. Genet..

[43] Arya R, Spaeth C, Gilbert DL, Leach JL, Holland KD (2017). GNAO1-associated epileptic encephalopathy and movement disorders: c.607G>A variant represents a probable mutation hotspot with a distinct phenotype. Epileptic. Disord..

[44] Saitsu H (2016). Phenotypic spectrum of GNAO1 variants: epileptic encephalopathy to involuntary movements with severe developmental delay. Eur. J. Hum. Genet..

[45] Kest B (2009). Gnao1 (G alphaO protein) is a likely genetic contributor to variation in physical dependence on opioids in mice. Neuroscience.

[46] Lehrmann E (2006). Transcriptional changes common to human cocaine, cannabis and phencyclidine abuse. PLoS. One.

[47] Caloca MJ, Wang H, Kazanietz MG (2003). Characterization of the Rac-GAP (Rac-GTPase-activating protein) activity of beta2-chimaerin, a ‘non-protein kinase C’ phorbol ester receptor. Biochem. J..

[48] Barrio-Real L, Barrueco M, Gonzalez-Sarmiento R, Caloca MJ (2013). Association of a novel polymorphism of the beta2-chimaerin gene (CHN2) with smoking. J. Invest. Med..

[49] Hao L, Luo T, Dong H, Tang A, Hao W (2017). CHN2 promoter methylation change may be associated with methamphetamine dependence. Shanghai Arch. Psychiatry.

[50] Davies SJ (2004). Mapping of three translocation breakpoints associated with orofacial clefting within 6p24 and identification of new transcripts within the region. Cytogenet. Genome Res..

[51] Vozza A (2017). Biochemical characterization of a new mitochondrial transporter of dephosphocoenzyme A in Drosophila melanogaster. Biochim. Biophys. Acta Bioenerg..

[52] Zador, F. et al. Kynurenines and the Endocannabinoid System in Schizophrenia: Common Points and Potential Interactions. *Molecules* **24**, 3709 (2019). 10.3390/molecules24203709 PMC6832375 31619006

[53] Toth, F., Cseh, E. K. & Vecsei, L. Natural Molecules and Neuroprotection: Kynurenic Acid, Pantethine and alpha-Lipoic Acid. *Int. J. Mol. Sci.* **22**, 403 (2021). 10.3390/ijms22010403 PMC7795784 33401674

[54] Steuer, A. E. et al. Comparative Untargeted Metabolomics Analysis of the Psychostimulants 3,4-Methylenedioxy-Methamphetamine (MDMA), Amphetamine, and the Novel Psychoactive Substance Mephedrone after Controlled Drug Administration to Humans. *Metabolites* **10**, 306 (2020). 10.3390/metabo10080306 PMC7465486 32726975

[55] Kahlig KM (2005). Amphetamine induces dopamine efflux through a dopamine transporter channel. Proc. Natl Acad. Sci. USA.

[56] Fagen ZM, Mitchum R, Vezina P, McGehee DS (2007). Enhanced nicotinic receptor function and drug abuse vulnerability. J. Neurosci..

[57] Bamford NS (2008). Repeated exposure to methamphetamine causes long-lasting presynaptic corticostriatal depression that is renormalized with drug readministration. Neuron.

[58] Bierut LJ (2007). Novel genes identified in a high-density genome wide association study for nicotine dependence. Hum. Mol. Genet..

[59] Tobacco and Genetics Consortium. (2010). Genome-wide meta-analyses identify multiple loci associated with smoking behavior. Nat. Genet..

[60] Volkow N, Swanson JM (2008). Does childhood treatment of ADHD with stimulant medication affect substance abuse in adulthood?. Am. J. Psychiatry.

[61] Liu JZ (2010). Meta-analysis and imputation refines the association of 15q25 with smoking quantity. Nat. Genet..

[62] Harstad E, Levy S (2014). & Committee on Substance Abuse. Attention-deficit/hyperactivity disorder and substance abuse. Pediatrics.

[63] Humphreys KL, Eng T, Lee SS (2013). Stimulant medication and substance use outcomes: a meta-analysis. Jama. Psychiatry.

[64] Sulaiman AH (2014). The risk and associated factors of methamphetamine psychosis in methamphetamine-dependent patients in Malaysia. Compr. Psychiatry.

[65] Salo R (2011). Psychiatric comorbidity in methamphetamine dependence. Psychiatry Res..

[66] Rungnirundorn T, Verachai V, Gelernter J, Malison RT, Kalayasiri R (2017). Sex differences in methamphetamine use and dependence in a Thai treatment center. J. Addict. Med..

[67] Dong H (2017). Comparison of demographic characteristics and psychiatric comorbidity among methamphetamine-, heroin- and methamphetamine-heroin co-dependent males in Hunan, China. BMC Psychiatry.

