# Supplementary material for: Genome-wide association study of stimulant dependence
Source: Transl Psychiatry. 2021 Jun 29;11:363. doi: 10.1038/s41398-021-01440-5 (PMC8257618; doi:10.1038/s41398-021-01440-5)

**Supplementary Figure 1.** Quantile-quantile plots for the GWAS in **(A)** African American (AA), **(B)** European ancestry (EA), and **(C)** combined AA and EA datasets. Estimates of p-value inflation (λ) are 1.020, 1.038, and 1.041 for the GWAS in AAs, EAs and combined population, respectively. Red dots represent the observed genome-wide significant variants.

(a)

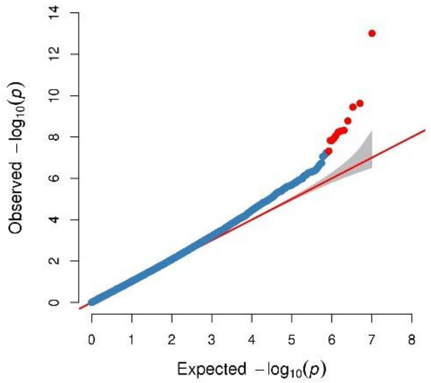


(b)

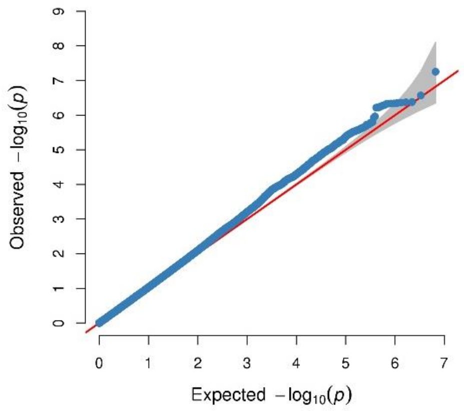


(c)

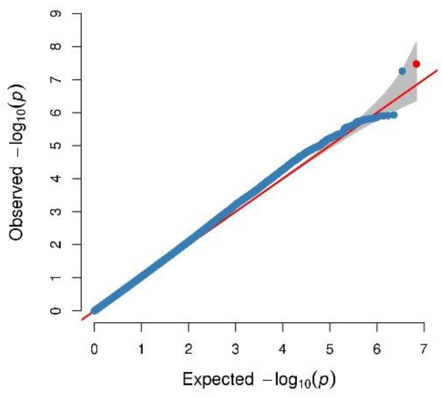

**Supplementary Figure 2**. GWAS results for **(A)** African American (AA), **(B)** European ancestry (EA), and **(C)** combined AA and EA discovery datasets. Red dots represent SNPs that surpassed the genome-wide significant threshold ($p<1\times10^{-8}$) indicated by a red line. Green dots above the green line represent SNPs that showed suggestive evidence for association ($p<1\times10^{-6}$).

**(A)**

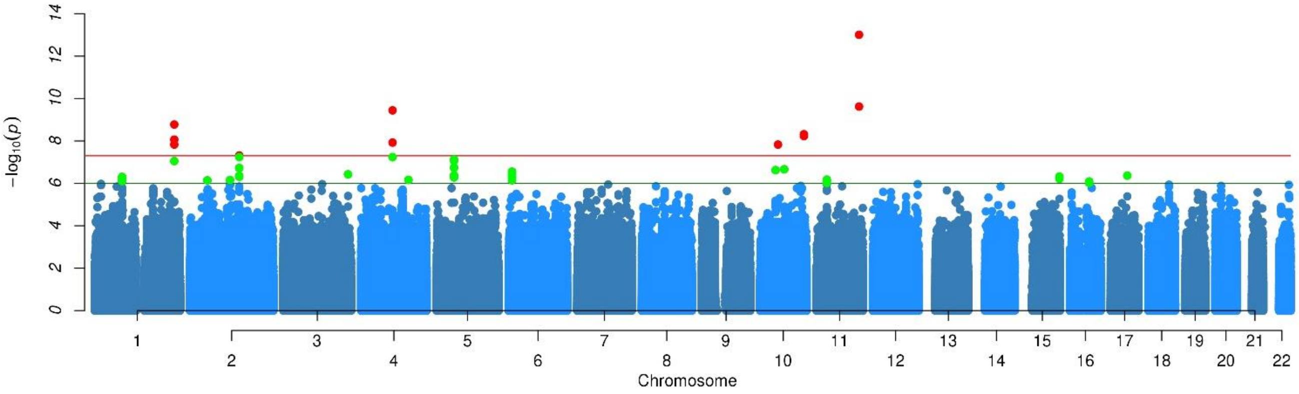


(B)

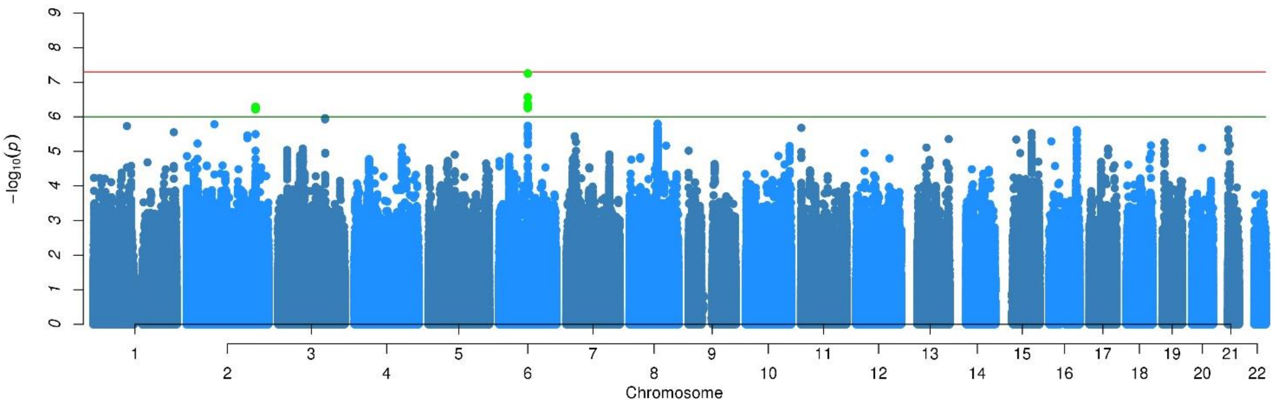


(C)

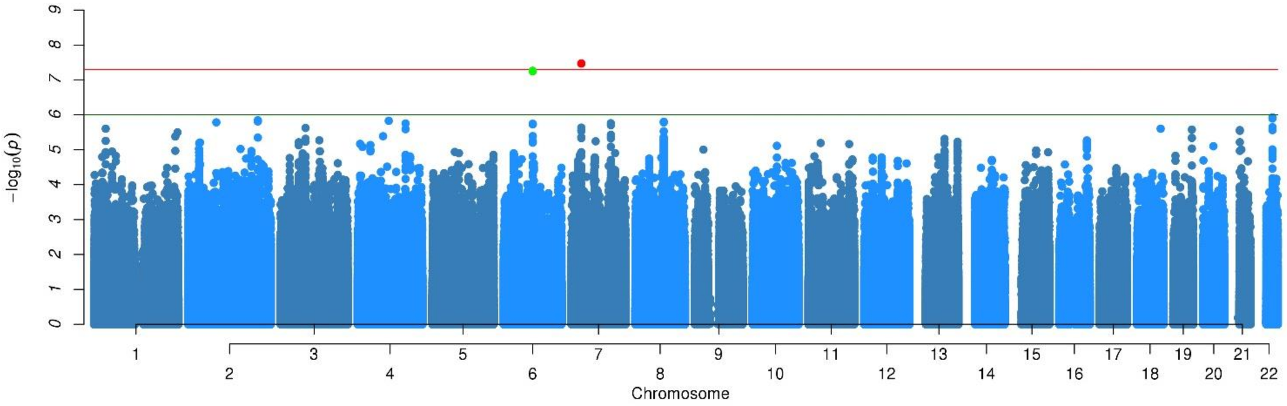

Supplement: Supplementary file 1 — Supplementary Figures [file 41398_2021_1440_MOESM1_ESM.pdf]
